# Epigenetic Landscapes of Pain: DNA Methylation Dynamics in Chronic Pain

**DOI:** 10.3390/ijms25158324

**Published:** 2024-07-30

**Authors:** Huan-Yu Xiong, Arne Wyns, Jente Van Campenhout, Jolien Hendrix, Elke De Bruyne, Lode Godderis, Siobhan Schabrun, Jo Nijs, Andrea Polli

**Affiliations:** 1 Pain in Motion Research Group (PAIN), Department of Physiotherapy, Human Physiology and Anatomy, Faculty of Physical Education & Physiotherapy, Vrije Universiteit Brussel, 1090 Brussels, Belgium; huanyu.xiong@vub.be (H.-Y.X.); arne.wyns@vub.be (A.W.); jente.van.campenhout@vub.be (J.V.C.); jolien.hendrix@vub.be (J.H.); andrea.polli@vub.be (A.P.); 2 Department of Public Health and Primary Care, Centre for Environment & Health, KU Leuven, 3000 Leuven, Belgium; lode.godderis@kuleuven.be; 3 Research Foundation—Flanders (FWO), 1000 Brussels, Belgium; 4 Translational Oncology Research Center (TORC), Team Hematology and Immunology (HEIM), Vrije Universiteit Brussel, 1090 Brussels, Belgium; elke.de.bruyne@vub.be; 5 The School of Physical Therapy, University of Western Ontario, London, ON N6A 3K7, Canada; sschabru@uwo.ca; 6 The Gray Centre for Mobility and Activity, Parkwood Institute, St. Joseph’s Healthcare, London, ON N6A 4V2, Canada; 7 Chronic Pain Rehabilitation, Department of Physical Medicine and Physiotherapy, University Hospital Brussels, 1090 Brussels, Belgium; 8 Department of Health and Rehabilitation, Unit of Physiotherapy, Institute of Neuroscience and Physiology, Sahlgrenska Academy, University of Gothenburg, 41390 Göterbog, Sweden

**Keywords:** DNA methylation, demethylation, chronic pain, central sensitization

## Abstract

Chronic pain is a prevalent condition with a multifaceted pathogenesis, where epigenetic modifications, particularly DNA methylation, might play an important role. This review delves into the intricate mechanisms by which DNA methylation and demethylation regulate genes associated with nociception and pain perception in nociceptive pathways. We explore the dynamic nature of these epigenetic processes, mediated by DNA methyltransferases (DNMTs) and ten-eleven translocation (TET) enzymes, which modulate the expression of pro- and anti-nociceptive genes. Aberrant DNA methylation profiles have been observed in patients with various chronic pain syndromes, correlating with hypersensitivity to painful stimuli, neuronal hyperexcitability, and inflammatory responses. Genome-wide analyses shed light on differentially methylated regions and genes that could serve as potential biomarkers for chronic pain in the epigenetic landscape. The transition from acute to chronic pain is marked by rapid DNA methylation reprogramming, suggesting its potential role in pain chronicity. This review highlights the importance of understanding the temporal dynamics of DNA methylation during this transition to develop targeted therapeutic interventions. Reversing pathological DNA methylation patterns through epigenetic therapies emerges as a promising strategy for pain management.

## 1. Introduction

Chronic pain is a primary reason individuals seek medical care, accounting for severe disability and socio-economic burdens worldwide [1]. Among the leading causes of years lost to disability, three (back pain, musculoskeletal disorders, and neck pain) are chronic pain conditions [2]. Affecting an estimated 11% to 40% of the global population [3,4], the costs for chronic pain are much larger than the annual costs of heart disease, cancer, and diabetes combined [5]. The lack of precise diagnostic tools and effective treatment options often leads to frustration for both patients and clinicians [6], with some patients developing resistance to conventional opioid analgesics [7]. Furthermore, existing strategies for pain prevention and management are largely inadequate and unsatisfactory, leading to a reduced quality of life.

Ground-breaking research has led the World Health Organization to recognize chronic pain as a disease characterized by intricate functional and structural alterations in the brain, neuroinflammation, and increased sensitivity of the central nervous system (CNS) to nociceptive stimuli [8,9,10,11,12]. This condition is further complicated by aberrant gene expression within neural cells responsible for nociceptive signal processing [12,13]. While genetic mutations provide some insight, the emerging field of epigenetics offers a more dynamic understanding by clarifying gene–environment interactions and gene expression patterns associated with chronic pain [14].

Epigenetic mechanisms refer to a series of physiological processes for stable control over gene expression to establish tissue- and cell-specific phenotypes [15]. DNA methylation, a key epigenetic mechanism, allows for dynamic and reversible gene regulation without altering the DNA sequence. Pathological DNA methylation patterns, observed in conditions such as cancer, neurological disorders, and CNS dysregulation, can lead to aberrant gene expression, thereby affecting pathogenesis [16,17,18]. Recent findings indicate that DNA methylation modulates the expression of pro-nociceptive and anti-nociceptive genes in the nociceptive pathways [19,20,21]. Patients with chronic pain syndromes, such as fibromyalgia [22,23], chronic widespread pain [24,25], chronic low back pain (CLBP) [26,27], migraine [28], and chronic postoperative pain [29,30], display altered global DNA methylation profiles compared to healthy individuals. Moreover, specific gene/locus DNA methylation alterations have been correlated with pain hypersensitivity, neuronal hyperexcitability, central sensitization, and immune/inflammatory responses [20,31].

Epigenetics has become one of the most promising fields for unravelling the gene expression patterns of many diseases, leading to ground-breaking discoveries and innovative therapies, including novel cancer treatments [32,33]. Chronic pain is also considered a reversible process substantially shaped by the interplay between noxious stimuli and the neuropsychological environment [34]. Therefore, investigating the role of DNA methylation in pain progression and identifying biomarker signatures closely related to chronic pain onset and evolution are imperative [35].

Here, this narrative review offers an up-to-date exploration of the potential involvement of DNA methylation in the pathogenesis and symptomatology of chronic pain. We highlight how DNA (de)methylation modifications regulate genes associated with nociception and pain perception in pain circuitry. By evaluating DNA methylation patterns as potential diagnostic markers for chronic pain, we underscore the need for comprehensive strategies to unravel the complex transition from acute to chronic pain. In contrast to previous reviews, this review addresses the scarcity of clinical studies involving human subjects with chronic pain, providing a comprehensive overview of the current landscape in chronic pain research and potential therapeutic avenues. Finally, we summarize unresolved questions in pain epigenetics, emphasizing areas that require further investigation.

## 2. Search Strategy and Selection Criteria

A comprehensive search of peer-reviewed articles was completed to outline the dynamics of DNA methylation under chronic pain conditions. We searched the PubMed, MEDLINE, Embase, and Web of Science databases, considering publications up to June 2024. The aim was to identify randomized clinical trials, observational studies, preclinical studies, and narrative reviews in English for inclusion in the text and tables. The literature search used the following terms: DNA methylation, demethylation, DNA methyltransferase, ten-eleven translocation, Methyl-CpG-binding domain, pain, and pain model. The search was limited to English-language publications. A preliminary screening of articles was conducted based on their title and abstract to ensure relevance. This was followed by a thorough screening based on inclusion criteria and quality assessment. Additionally, reference lists of retrieved articles were examined to identify potentially relevant studies not found in the initial search, further supplementing the discussion. The included studies focused on primary clinical research on DNA methylation in human subjects with various chronic pain conditions and preclinical studies on DNA methylation in animal pain models with specific investigation time points.

## 3. DNA Methylation Dynamics in Chronic Pain

DNA methylation involves the transfer of a methyl group (-CH₃) from S-adenosyl-methionine (SAM) to the fifth carbon of the cytosine ring, resulting in the formation of 5-methylcytosine (5-mC) [36]. This modification primarily occurs at cytosine-phosphate-guanosine (CpG) sites of double-stranded DNA, which tend to cluster into ‘CpG islands’—regions often encompassing gene promoters [37]. Around 75% of protein-encoding genes in humans present this enrichment at their promoters [38]. The influence of DNA methylation on transcriptional activity is profound and location-dependent. In promoter regions, DNA methylation generally suppresses gene expression by hindering transcription factor binding, recruiting transcription repressors, and prompting chromatin remodelling. Consequently, this modification serves as a transcriptional barrier, preventing RNA polymerase from initiating gene transcription. Indeed, DNA methylation fulfils two essential roles in maintaining genomic integrity: (i) silencing potentially harmful elements such as transposons, viral DNA, and genes that should not be expressed; and (ii) preventing transcription factor binding to specific sites in promoter regions or recruiting transcription repressors.

The intricate relationship between DNA methylation and gene expression is a cornerstone of genomic regulation. Notably, the orchestration of DNA methylation patterns is managed by a series of specialized enzymes, each with a distinct function: (i) writers: enzymes that add the modifications to the DNA, establishing the methylation landscape; (ii) erasers: enzymes that remove the methyl marks, allowing for the dynamic regulation of gene expression; and (iii) readers: enzymes that identify and interpret the methylation patterns, translating methylation marks into biological outcomes.

### 3.1. DNMTs: Writers of DNA Methylation in Pain Chronicity

**Figure 1:**
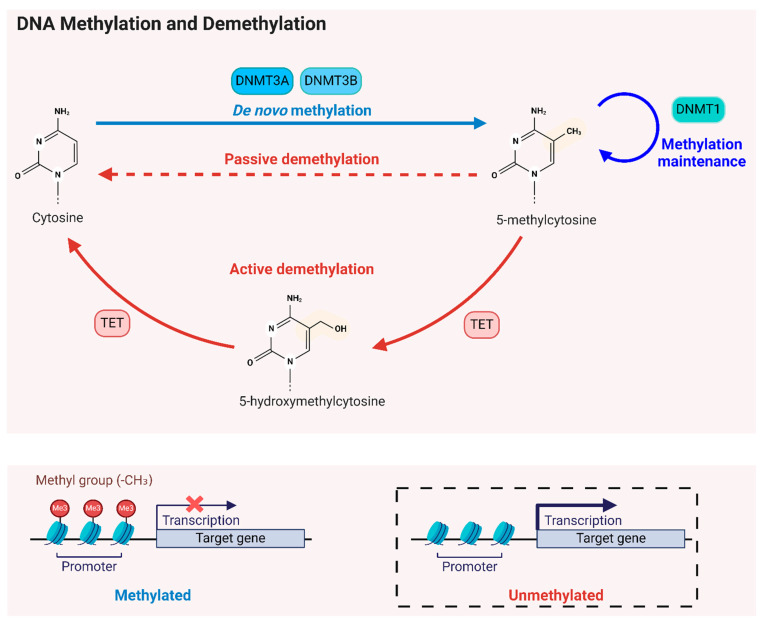
DNA methylation and demethylation modifications. DNA methylation and demethylation are regulated by several key enzymes. de novo DNMTs (DNMT3a and DNMT3b) introduce new methylation marks to previously unmethylated DNA, while maintenance DNMT (DNMT1) perpetuates existing methylation patterns and facilitates their repair. TET enzymes convert 5-mC to 5-hydroxymethylcytosine (5-hmC) within the CpG dinucleotide, initiating active DNA demethylation. Passive DNA demethylation occurs during DNA replication, resulting in the gradual removal of methylation marks if maintenance of methylation on hemi-methylated DNA is hindered, such as by reduced DNMT activity or a deficiency of SAM (methyl donor). When methylation occurs at CpG islands in gene promoters, it generally represses gene expression. The removal of methyl groups from CpG sites can lead to the activation of gene expression.

DNA methyltransferases (DNMTs) are responsible for adding methyl groups to cytosine nucleotides in DNA, resulting in 5-mC [39,40]. The DNMT family comprises two main classes: the de novo DNMTs (DNMT3a and DNMT3b), which introduce new methylation marks to previously unmethylated DNA, and the maintenance DNMT (DNMT1), which perpetuates already established methylation patterns and facilitates their repair [41,42] (Figure 1). During DNA replication, DNMT1 recognizes hemi-methylated DNA and ensures the new strand is appropriately methylated, thus preserving the epigenetic information through cell division and in response to DNA damage [43].

Recent studies have implied a pro-nociceptive role of DNMTs in almost all major stations in the nociceptive pathway, including the dorsal root ganglion (DRG) [44,45], spinal cord [46,47], and brain regions such as the amygdala [48] and prefrontal cortex (PFC) [49], thereby contributing to chronic pain. Alterations in DNA methylation patterns at these stations can significantly influence gene expression, thereby affecting an individual’s normal physiological functions and processing of pain.

In neuropathic pain models, significant upregulations of DNMT1 [50] and DNMT3a [51] in DRG neurons were correlated with nociceptive hypersensitivity. These upregulations are associated with DNA methylation alteration at the Kcna2 promoter, leading to the epigenetic silencing of the Kcna2 gene, which encodes the Kv1.2 potassium channel. Reduced Kv1.2 levels diminish voltage-dependent potassium currents, depolarize the resting membrane potential, and promote neuronal hyperexcitability in DRG neurons, thereby amplifying spinal cord sensitization and neuropathic pain manifestations [52,53]. Moreover, DNMT3a-mediated epigenetic silencing of Kv1.2 in the spinal dorsal horn is also implicated in bone cancer pain [54].

In addition, a study demonstrated that DNMT3a-mediated downregulation of the K2p1.1 gene might contribute to paclitaxel-induced neuropathic pain [55]. Paclitaxel, a chemotherapy drug often used in ovarian cancer, induces substantial transcriptional changes in DRG neurons, leading to chemotherapy-induced neuropathic pain [56,57]. Following paclitaxel treatment, DNMT3a levels rise while K2p1.1 mRNA and protein levels reduce, resulting in a decrease in outward potassium currents and an increase in DRG neuron excitability [58], which ultimately lead to mechanical allodynia and thermal hyperalgesia. Conversely, inhibition of DNMT3a via RG108, a specific non-nucleoside DNMT inhibitor, significantly reduces paclitaxel-induced nociceptive hypersensitivity, suggesting the pro-nociceptive role of DNMT3a in neuropathic pain development [55].

Furthermore, DNMT3a upregulation in the spinal cord [47] and DRG [59] following peripheral nerve injury also orchestrate the epigenetic silencing of the the mu-1 opioid receptor gene (Oprm1) gene, which encodes the mu-opioid receptor (MOR). MOR’s presence on presynaptic axon terminals in the spinal cord reduces neurotransmitter release by inhibiting voltage-gated calcium channels, thereby attenuating nociceptive input to the CNS [60]. MOR is also located post-synaptically, where it activates G protein-coupled inwardly, rectifying potassium channels and reducing neuronal excitability [60]. Moreover, it is well established that decreased MOR protein levels reduce the efficacy of opioid analgesics [61,62]. Consistent with this, inhibition of DNMT3a in vivo can prevent the spinal nerve ligation (SNL)-induced DNA methylation of Oprm1 in the DRG, restore the analgesic effects of morphine or loperamide, and reduce the development of analgesic tolerance [59].

Notably, Jiang et al. [63,64] reported that DNMT3b downregulation following SNL prevents DNA methylation maintenance, leading to decreased DNA methylation levels of the G-protein-coupled receptor 151 (GPR151) and chemokine receptor CXCR3 genes, significantly increasing their expression in spinal neurons. The upregulation of these genes may contribute to neuropathic pain through activation of the mitogen-activated protein kinase (MAPK) signalling pathway [65]. In addition, in inflammatory pain models, significant hyperalgesia is observed, which correlates with increased CXCR4 mRNA/protein expression in the DRG [66]. This upregulation pattern is paralleled by significant demethylation at the CXCR4 gene promoter and a decrease in DNMT3b levels in the DRG.

### 3.2. MBD: Readers of DNA Methylation in Pain Modulation

Methyl-CpG-binding domain (MBD) proteins are key interpreters of DNA methylation, selectively recognizing and binding to methylated CpG sites within the genome. This binding is a critical step in transducing the methylation signal, which in turn regulates gene expression. MBD proteins achieve this by recruiting proteins with chromatin remodelling capacities, transcription factors, or repressors to methylated DNA regions [67,68]. At promoter regions, these recruited factors often modify chromatin structure into a more condensed state, leading to transcriptional repression. Therefore, MBD proteins are essential for the regulation of gene activity that is initiated by DNA methylation, and can function as either a transcriptional activator or repressor depending on their protein partners and target genes [69].

In neuropathic pain models, MBD1-deficient mice display reduced responses to acute sensory stimuli after nerve injury [70]. Correspondingly, MBD1-deficient mice show increased levels of Oprm1 and Kcna2 in the DRG. In contrast, MBD1 overexpression in the DRG induces spontaneous pain and evoked nociceptive hypersensitivity in MBD1-deficient mice. This effect is likely due to MBD1’s suppression of Oprm1 and Kcna2 expression, potentially downregulating MOR and Kv1.2 proteins in nociceptive pathways [51,70]. Supporting this, acute knockout of MBD1 via siRNA-MBD1 boosts MOR and Kv1.2 protein abundance, reducing pain sensitivity [70].

Previous studies have also reported a potential pro-nociceptive role of Methyl-CpG-binding protein 2 (MeCP2) in nociceptive pathways [71]. For example, mutations in the MeCP2 gene cause Rett syndrome [72], where decreased pain perception is commonly reported [73,74]. Moreover, an increase in both global DNA methylation and MeCP2 expression in the spinal cord two weeks after chronic constriction injury (CCI) correlates with mechanical allodynia and thermal hyperalgesia [75]. Conversely, inhibition of DNA methylation through 5-azacytidine treatment can reverse these effects, reducing CCI-induced hyperalgesia [75,76]. In addition, MeCP2 mutant mice also show reduced nociceptive sensitivity [71]. These findings indicate that a reduction in functional MeCP2 could contribute either directly or indirectly to reduced nociceptive sensitivity.

Recent studies have however challenged this view, indicating that MeCP2 expression is reduced in a cell-type-specific manner after nerve injury [77,78,79], and MeCP2-knockout mice show hypersensitivity to noxious stimuli [80]. Increased MeCP2 expression attenuated spared nerve injury (SNI)-induced mechanical and thermal hypersensitivity, suggesting that it weakens the development of neuropathic pain [81]. Additionally, MeCP2 deletion in peripheral sensory neurons causes nociceptive hypersensitivity, likely due to the loss of gamma-aminobutyric acid (GABA) receptors on sensory neuron terminals and subsequent diminished presynaptic inhibition. This loss may directly increase neuronal excitability, potentially promoting central sensitization within nociceptive pathways [77].

### 3.3. TET: Erasers of DNA Methylation in Chronic Pain

DNA methylation typically suppresses gene transcription, while DNA demethylation can (re-)activate it through either active or passive processes [82,83]. The ten-eleven translocation (TET) enzymes—including TET1, TET2, and TET3—function as ‘erasers’ of DNA methylation by converting 5-mC to 5-hydroxymethylcytosine (5-hmC) within the CpG dinucleotide, thereby initiating active DNA demethylation [84,85]. Passive DNA demethylation, on the other hand, occurs during DNA replication and can lead to the gradual removal of the methylation marks over time if the maintenance of methylation on hemi-methylated DNA is hindered, such as by reduced DNMT activity or a deficiency of SAM (methyl donor) [86]. Unlike active DNA demethylation, which is an enzymatic process that can occur independently of DNA replication, passive demethylation does not require direct enzymatic action [87]. 5-hmC markers are widely distributed across mammalian DNA, and TET enzymes are expressed in diverse tissues, including the brain and blood [88].

Since their discovery, TET enzymes have become a central focus of pain epigenetics research. In models of osteoarthritis [89], visceral pain [90], inflammatory pain [91,92], and neuropathic pain [93,94,95], DNA demethylation patterns modulated by TET enzymes are associated with nociceptive hypersensitivity and allodynia. An increase in TET expression and 5-hmC enrichment in the spinal cord and DRG post-injury highlights the link between TET-mediated demethylation and the onset of chronic pain. Moreover, intrathecal administration of a TET1 vector induces long-lasting allodynia and hyperalgesia in naive rats, starting on day 7, peaking at day 14, and lasting for at least 4 weeks [96]. Conversely, TET1 knockdown mitigates established pain behaviours and downregulates TET1 in dorsal horn neurons.

Notably, several studies have identified alterations in specific gene expressions due to DNA demethylation regulation, suggesting a potential role in chronic pain. For example, in neuropathic pain models, SNL enhances TET1 expression in dorsal horn neurons, leading to 5-hmC enrichment at the brain derived neurotrophic factor (BDNF) promoter, which in turn increases spinal BDNF expression and neuronal excitability [97]. Moreover, one week after SNL, TET1 overexpression also hinders DNMTs (DNMT1, DNMT3A, and DNMT3B) from binding to the BDNF promoter, thereby preventing transcriptional silencing via DNA methylation. Conversely, knockdown of Tet1 not only decreased TET1 binding and 5-hmC enrichment at the BDNF promoter, but also reduced BDNF expression and alleviated allodynia and nociceptive hypersensitivity [97]. Hence, spinal TET1 enhances BDNF transcription by promoting TET1-mediated demethylation and inhibiting DNMT-dependent methylation. In addition, inhibiting TET enzymes, such as TET1 and TET2, significantly reduces nociceptive behaviours by blocking the upregulation of Stat3 [92] and NLRP3 [95] expression, respectively.

However, in another neuropathic pain model, TET1 overexpression via full length-TET1 mRNA microinjection into the DRG significantly alleviated SNL-induced nociceptive hypersensitivity during both development and maintenance phases without affecting acute pain [98]. This intervention restores morphine analgesia and reduced morphine tolerance following SNL. Moreover, Tet1 overexpression rescues the expression of MOR and Kv1.2 by decreasing 5-mC and enhancing 5-hmC levels at the Oprm1 (encoding MOR) and Kcna2 (encoding Kv1.2) promoters in the DRG. Peripheral nerve injury is known to suppress MOR and Kv1.2 expression by DNA methylation-mediated epigenetic silence at these sites, thereby contributing to neuropathic pain. TET1-mediated DNA demethylation may counteract this effect.

## 4. Altered DNA Methylation Patterns in Patients with Chronic Pain: DNA Hypermethylation and Hypomethylation

While the previous parts of this narrative review were based on preclinical studies, this third section focusses on epigenetic studies in patients with chronic pain conditions (Table 1). Advancements in genome-scale mapping have revolutionized our ability to evaluate DNA methylation levels and sites under various conditions. Unbiased genome-wide analyses through digital restriction enzyme analysis of methylation (DREAM) and reduced representation bisulfite sequencing (RRBS) have revealed dynamic DNA methylation changes in many chronic pain disorders. These techniques overcome potential biases and increase the accuracy of methylation quantitative measurements, facilitating the detection of differentially methylated regions (DMRs) and cytosines (DMCs) that could serve as biomarkers for chronic pain. DNA extracted from peripheral blood samples can be used to develop non-invasive molecular tests, as methylation analysis in peripheral blood cells often reflects methylation levels in the target tissue [99,100], as supported by evidence of blood–brain methylation correspondence [101,102]. For example, specific overlapping DNA methylation signatures of chronic pain have been found in both the PFC and peripheral T cells [103].

The interplay between genes and the environment is pivotal in chronic pain. Investigations into global DNA methylation differences between chronic pain patients and healthy individuals have scrutinized methylation patterns of pain-related genes to identify diagnostic and therapeutic markers. For instance, studies involving patients with fibromyalgia identified 1610 promoter regions with differentially methylated CpG sites, most (69%) of which were hypomethylated compared to healthy controls [22,23,104]. In a large cohort study of twin pairs with and without chronic widespread pain as well as non-related individuals, with repeated measurements over three years, significant DNA methylation differences were observed between patients with chronic widespread pain and healthy subjects, with epigenetic factors accounting for 6% of the variance in the pain phenotype of chronic widespread pain [24]. This study highlights methylation enrichment in neurological pathways and the potential involvement of genes such as RE1-Silencing Transcription Factor (REST), Monoamine Oxidase B (MAOB), and Collagen Type I Alpha 2 Chain (COL1A2) in immune function, inflammatory responses, and central sensitization [25,105].

In patients with non-specific CLBP, 159 differentially methylated positions were identified, mostly hypomethylated [27]. Individuals who developed CLBP showed lower global DNA methylation levels than those with resolved acute low back pain or healthy controls, suggesting that DNA hypomethylation might contribute to pain chronicity [106]. Additionally, significant differences were observed between individuals with early vs. advanced intervertebral disc degeneration [107], with around 98% of CpG sites being hypermethylated in advanced stages, although their direct association with pain was not evaluated.

In addition, a large cohort study of patients with a period of 3–18 months post-traumatic limb amputation, revealed that those who developed complex regional pain syndrome (CRPS) had 48 differentially methylated CpG sites compared to those with non-CRPS neuropathic pain, with 85% being hypomethylated [108]. Together with the above-described study regarding the transition from acute to chronic low back pain, this highlights the potential diagnostic and therapeutic importance of global DNA methylation in the progression from acute to chronic pain.

Global DNA methylation serves as an indicator of the overall state of the DNA methylation machinery, influencing genome function and organization on a large scale [109]. In other diseases, such as cancer, altered DNA methylation landscapes affect thousands of genes [110] and intergenic regions [111]. DNA methylation reprogramming involves both global changes in genome methylation and gene-specific alterations targeting discrete regulatory regions, thereby producing lasting effects on the chromatin structure, and transcriptome, ultimately influencing many aspects of cellular function.

In addition to these global changes, recent clinical studies have identified transcriptional changes in specific genes due to DNA methylation in chronic pain sufferers. Pro- and anti-nociceptive genes, which promote or inhibit pain sensation, respectively, are particularly prone to DNA methylation changes. For instance, a positive correlation has been established between a DNA hypermethylation pattern of the transient receptor potential ankyrin 1 (TRPA1) and pain symptoms in patients with CLBP, postherpetic neuralgia, and Crohn’s disease [26,112,113]. The TRPA1 channel, known for detecting various noxious stimuli [114], is increasingly recognized as a key player in nociceptive modulation [115]. DNA methylation alterations of the tumor necrosis factor (TNF) gene may influence the risk of developing chronic breast pain in patients with breast cancer undergoing surgery [29]. Additionally, increased DNA methylation of the secreted protein, acidic and rich in cysteine (SPARC) gene, and its subsequent downregulation, have been observed in animal models and patients with CLBP [116]. SPARC, a matricellular protein important for tissue remodelling and injury response [117], may have its activity and gene transcription repressed through DNA hypermethylation, potentially contributing to the development of chronic pain.

**Table 1:** DNA methylation reprogramming in patients with chronic pain.

Pain Conditions	Study Design	Tissue	Global DNA Methylation	Specific Genes	Functional Genomic Analyses Methods	Functional Enrichment Analysis	Reference
Acute low back pain (n = 14), Chronic low back pain (n = 15), Healthy controls (n = 16)	Cross-sectional	Blood tissue	DNA hypomethylation (genome-wide)	BDNF, CX3CR1, TNF, and many others	\	\	[106]
Non-specific chronic low back pain (n = 50), Healthy controls (n = 48)	Cross-sectional	Blood tissue	DNA hypomethylation (genome-wide)	CELSR1, KIF11, NAV1, and many others	Gene Ontology (GO) pathway enrichment analyses	Immune signalling, endochondral ossification, and G-protein-coupled transmissions	[27]
Early-stage disc degeneration (n = 8), Advanced-stage disc degeneration (n = 8)	Cohort study	Nucleus pulpous tissues from intervertebral disc	DNA hypermethylation (genome-wide)	CARD14, GNL3, MAPKAPK5, and many others	Gene Ontology (GO) pathway enrichment analyses	Hemophilic cell adhesion and cell–cell adhesion	[107]
Fibromyalgia (n = 42), Healthy controls (n = 42)	Cross-sectional	Blood tissue	DNA hypermethylation (genome-wide)	GCSAML, GRM2, TRPA1, and many others	\	\	[105]
Fibromyalgia (n = 24), Healthy controls (n = 24)	Cross-sectional	Blood tissue	DNA hypomethylation (genome-wide)	\	MetaCore network analysis	MAPK signalling pathway and actin cytoskeleton regulation	[23]
Fibromyalgia (n = 10), Healthy controls (n = 42)	Cross-sectional	Blood tissue	DNA hypermethylation (genome-wide)	BDNF, NAT15, HDAC4, and many others	Gene Ontology (GO) pathway enrichment analyses	Neuron differentiation and nervous system development	[22]
Chronic widespread pain (n = 50), Twins without chronic widespread pain (n = 50), Healthy controls (n = 1608)	Cohort study	Blood tissue	\	\	Gene Ontology (GO) pathway enrichment analyses	Neurological pathways	[24]
Chronic fatigue syndrome and comorbid fibromyalgia (n = 28), Healthy controls (n = 26)	Cross-sectional	Blood tissue	DNA hypomethylation (gene-specific)	BDNF	\	\	[20]
Chronic fatigue syndrome and comorbid fibromyalgia (n = 28), Healthy controls (n = 26)	Cross-sectional	Blood tissue	DNA hypermethylation (gene-specific)	COMT	\	\	[31]
Chronic nociceptive pain (n = 18), Chronic neuropathic pain (n = 19), Healthy controls (n = 20)	Cohort study	Blood tissue	\	RAB10, RBFOX-1/CGRP, MAGI2, and many others	STRING (https://string-db.org/)	Neuro-musculoskeletal system, immune response, and inflammation	[118]
Knee osteoarthritis pain (n = 182), Healthy controls (n = 31)	Cross-sectional	Blood tissue	DNA hypermethylation (genome-wide)	RNF39, KCNC1, ZFP57, and many others	Ingenuity Pathway Analysis	Immune response and inflammation	[119]
Musculoskeletal pain (n = 20), Healthy controls (n = 9)	Cross-sectional	Blood tissue	DNA hypomethylation (genome-wide)	PM20D1, LCLAT1, GNE, and many others	Ingenuity Pathway Analysis	Immune response and GABA receptor signalling	[120]
Complex regional pain syndrome (n = 8), Neuropathic pain (n = 38)	Cross-sectional	Blood tissue	DNA hypomethylation (genome-wide)	COL11A1, GPR75, GPR75, and many others	Gene Ontology (GO) pathway enrichment analyses	Immune function	[108]
Multisomatoform disorder (n = 136), Healthy controls (n = 145)	Cohort study	Blood tissue	DNA hypomethylation (gene-specific)	Leptin promoter	\	\	[121]
Chronic back pain or postherpetic neuralgia (n = 12)	Observational study	Blood tissue	DNA hypermethylation (gene-specific)	TRPA1	\	\	[26]

## 5. Long-Lasting Dynamic DNA Methylation Reprogramming: The Key to the Transition from Acute to Chronic Pain States?

Recent preclinical studies have revealed rapid DNA methylation reprogramming in response to peripheral nerve injury, suggesting a potential causal role in the transition from acute to chronic pain [44,49] (Table 2). These DNA methylation alterations persist as transition occurs, indicating the significance of DNA methylation in the development of chronic pain. For instance, in neuropathic pain models, SNL upregulates TET1 expression in dorsal horn neurons. This leads to 5-hmC enrichment at the BDNF promoter, which in turn increases spinal BDNF expression and subsequent neuronal excitability [97]. TET1 upregulation begins on day 3 post-SNL, peaks at day 7, and persists through days 14 and 21. Correspondingly, SNL induces nociceptive hypersensitivity at these time points, mirroring the pattern of TET1 expression enhancement. Similarly, intrathecal administration of a TET1 vector in naive rats triggers long-lasting allodynia and hyperalgesia, starting on day 7, peaking at day 14, and lasting for at least four weeks [96]. Notably, increased global DNA methylation and MeCP2 expression in the spinal cord two weeks following CCI also correlate with mechanical allodynia and thermal hyperalgesia [75]. However, critical unanswered questions concern the origin and long-term temporal progression of these alterations, as well as their external validity to humans suffering from (chronic) neuropathic pain.

To track epigenetic reprogramming in acute and chronic pain models, several studies have examined DNA methylation profiles in the brain over time, from one day to one year after SNI [49,122,123]. Initially, DNA methylation reprogramming in the PFC begins as early as day 1 post-injury, indicating that methylation alterations precede chronic pain manifestation [49]. The PFC, part of cortical and subcortical networks activated during pain experiences, undergoes structural and functional changes over time in chronic pain [124], including grey matter loss, reorganization of synaptic connections, and gene expression modulation [125,126,127,128,129,130]. Six months after SNI, hyperalgesia and increased anxiety were accompanied by a DNA hypomethylation pattern in the PFC and amygdala but not in the visual cortex and thalamus [122,123]. Furthermore, SNI triggers a series of dynamic DNA methylation changes that persist up to one-year post-injury in the PFC, suggesting that these alterations are concurrent with the onset and establishment of chronic pain phenotypes [49].

During this period, hundreds of differentially methylated genes and gene ontologies were identified, emphasizing the need to consider the time point-specific differential methylation of pain-related genes [49,122,123]. Most pain-related genes exhibit differential methylation at a single time point, indicating a time-dependent recruitment or dysregulation of normal functions. These genes influence pain modulation across several domains such as ion channel activity, inflammatory responses, and neurotransmitter regulation. While some genes may be relevant only at a specific time point, persistent differentially methylated genes could embed a genomic memory of the initial injury, necessary for the development of chronic pain, and can influence long-term gene regulation associated with chronic pain [131].

The temporal dynamics of differential gene methylation and ontology enrichment highlight the significance of understanding these processes during the transition to chronic pain. At one day post-injury, differential DNA methylation is likely driven by tonic sensory afferent signalling and the systemic impact of peripheral inflammation. As pain progresses to a sub-chronic state two weeks post-injury, central mechanisms such as central sensitization and neuroinflammation trigger changes in synaptic signalling and neuron–glia interactions within nociceptive pathways. By three months and one-year post-injury under a chronic pain state, genes associated with immune function become predominant. Chronic pain is characterized by the dysregulation of descending inhibitory and facilitatory nociceptive pathways, persistent low-grade neuroinflammation, and maladaptive neuroplasticity. Epigenetic processes are particularly capable of influencing chromatin structure and transcription, thereby supporting lasting change and acting as mediators of adaptive processes. Indeed, epigenetic mechanism has been implicated in learning, memory, and pain, with DNA methylation reprogramming fitting well into this picture [14,132].

There are few human studies on specific time points and longitudinal changes in response to acute or chronic pain. To date, only one human study, spanning six months, has reported that individuals who developed CLBP exhibited lower global DNA methylation levels compared to those with resolved acute low back pain and healthy controls [106]. This suggests that DNA hypomethylation and subsequent changes in candidate gene expression may contribute to pain chronicity. These patterns may reflect underlying differences between acute, sub-chronic, and chronic pain, implying that therapeutic interventions might also vary in efficacy based on the duration of pain. Thus, it is imperative for future research to conduct longitudinal studies that investigate the temporal dynamics of DNA methylation among individuals suffering from various chronic pain conditions.

**Table 2:** DNA methylation modifications after injury in pain models.

Pain Models	Methylation Marker/Enzyme	When and Time Course	Tissue	DNA Methylation Regulation	Gene-Specific Expression Regulation	Inhibition (Inhibitors)	Nociceptive Behaviour Response to Inhibitors	Reference
SNI-induced neuropathic pain model	\	Starting on day 1, lasting for 1 year post-SNI	Prefrontal cortex	\	MAPKBP1, Icos, Unc5cl, and many others	\	\	[49]
Partial SNL-induced neuropathic pain model	DNMT3a ↓	30 days post-SNI	Amygdala	\	\	\	\	[48]
SNI-induced neuropathic pain model	\	9 months post-SNI	Prefrontal cortex, T cells	\	KCNAB3, KCNC4, DNMT1, and many others	\	\	[103]
SNI-induced neuropathic pain model	\	6 months post-SNI	Prefrontal cortex, amygdala	\	\	Environmental manipulation	Nociceptive hypersensitivity ↓	[123]
SNL-induced neuropathic pain model	DNMT3b ↓	Starting on day 1, lasting for 14 days post-SNL	Spinal dorsal horn	DNA hypomethylation (gene-specific)	GPR151 ↑ CXCR3 ↑	SiRNA-DNMT3b	Nociceptive hypersensitivity ↓	[63,64]
SNL-induced neuropathic pain model	DNMT3a ↑	7 days post-SNL	Spinal dorsal horn	DNA hypermethylation (gene-specific)	Kcna2 (Kv1.2) ↓	AAV5-Dnmt3a shRNA	Nociceptive hypersensitivity ↓	[51]
Bone cancer pain model	DNMT3a ↑	Starting on day 3, lasting for 12 days post	Spinal dorsal horn	DNA hypermethylation (gene-specific)	Kcna2 (Kv1.2) ↓	Decitabine	Nociceptive hypersensitivity ↓	[54]
SNL-induced neuropathic pain model	TET1 ↑ 5-hmC ↑	Starting on day 3, lasting for 21 days post-SNL	Spinal dorsal horn	DNA hypomethylation (gene-specific)	BDNF ↑	SiRNA-TET1	Nociceptive hypersensitivity ↓	[97]
CFA-induced inflammatory pain model	TET1 ↑ TET3 ↑ 5-hmC ↑	Starting on day 3, lasting for 14 days post-CFA	Spinal cord, Blood tissue	DNA hypomethylation (gene-specific)	STAT3 ↑	Lenti-T1-siRNA, Lenti-T3-siRNA	Nociceptive hypersensitivity ↓	[92]
Paclitaxel-induced neuropathic pain model	DNMT3a ↑	Starting on day 7, lasting for 21 days post-paclitaxel	DRG	DNA hypermethylation (gene-specific)	K2P.1.1 ↓	RG108	Nociceptive hypersensitivity ↓	[55]
SNL-induced neuropathic pain model	DNMT1 ↑	Starting on day 3, lasting for 14 days post-SNL	DRG	DNA hypermethylation (gene-specific)	Kcna2 (Kv1.2) ↓	RG108	Nociceptive hypersensitivity ↓	[50]
SNL-induced neuropathic pain model	DNMT3a ↑ MBD1 ↑	7 days post-SNL	DRG	DNA hypermethylation (gene-specific)	Oprm1 (MOR) ↓	ShRNA-DNMT3a	Nociceptive hypersensitivity ↓, Analgesic effects of morphine ↑, Analgesic tolerance of morphine ↓	[59]
CFA-induced inflammatory pain model	TET1 ↑	Starting on day 3, lasting for 14 days post-CFA	DRG	DNA hypomethylation (gene-specific)	TRPV1 ↑	Bobcat339 hydrochloride	Nociceptive hypersensitivity ↓	[91]
Bone cancer pain model	TET1 ↑	Starting on day 3, lasting for 21 days post-tumour cell inoculation	DRG	DNA hypomethylation (gene-specific)	TRPV4 ↑	Bobcat339 hydrochloride	Nociceptive hypersensitivity ↓	[133]
Diabetic neuropathic pain model	TET2 ↑	28 days post	DRG	DNA hypomethylation (gene-specific)	TXNIP ↑ NLRP3 ↑	SiRNA-TET2	Nociceptive hypersensitivity ↓	[95]
SNL-induced neuropathic pain model	MBD1 ↑	\	DRG	DNA Hypermethylation (gene-specific)	MOR and KV.1.2 ↓	SiRNA-MBD1	Nociceptive hypersensitivity ↓	[70]
SNI-induced neuropathic pain model	MeCP2 ↑	28 days post-SNI	DRG	DNA hypomethylation (gene-specific)	BDNF ↑	MeCP2-null	Nociceptive hypersensitivity ↓	[70]
Oral cancer pain model	\	\	DRG	DNA hypermethylation (gene-specific)	Oprm1 (MOR) ↓	Decitabine	Nociceptive hypersensitivity ↓	[134]

SNL, spinal nerve ligation; SNI, spared nerve injury; CFA, complete Freund’s adjuvant; siRNA, small-interfering RNA; DRG, dorsal root ganglion; DNMT, DNA methyltransferases; MBD, methyl-CpG-binding domain; MeCP2, methyl-CpG-binding protein 2; TET, ten-eleven translocation.

## 6. DNA Methylation Modification in Patients with Chronic Pain: Links to Central Sensitization

Chronic pain is not merely an extended version of acute pain; it involves complex and long-lasting structural and functional plasticity within the CNS [135]. These changes go beyond a simple pain–damage correlation, involving intricate forms of maladaptive plasticity at molecular, cellular, and systemic levels. Central sensitization, characterized by increased neuronal excitability, maladaptive neuroplasticity, and amplified response to nociceptive and non-nociceptive stimulus, is crucial in the pathogenesis of chronic pain [8]. The induction and maintenance of central sensitization depend on maladaptive alterations in the expression, distribution, and activity of ion channels, receptors, and inflammatory mediators [135,136]. Many of these long-lasting adaptations are sustained via the modulation of transcriptional responses, with DNA methylation—a key modulator of transcription—potentially contributing to these maladaptive processes in chronic pain.

**Figure 2:**
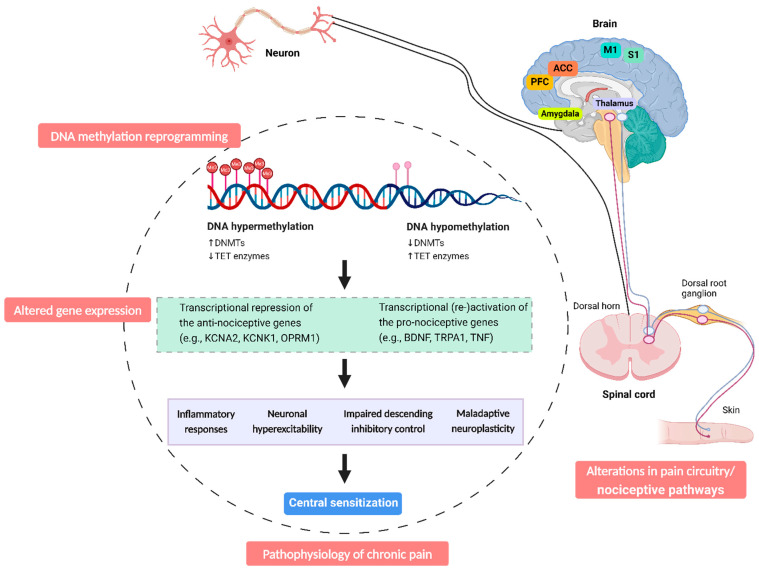
DNA methylation reprogramming in chronic pain. DNA methylation and demethylation are controlled by multiple enzymes. The expression levels of these enzymes can alter the methylation state of specific gene promoters. An increase in DNMTs facilitates hypermethylation, while a decrease in DNMT expression is associated with hypomethylation. Conversely, a reduction in TET proteins promotes hypermethylation, while increased TET expression can lead to hypomethylation. DNA hypermethylation typically leads to gene silencing, while hypomethylation in promoter regions generally results in gene activation. These changes in DNA methylation levels affect the expression of various pro- and anti-nociceptive genes, facilitating the long-lasting plasticity of the pain circuitry both centrally and peripherally, ultimately contributing to the development and maintenance of chronic pain. In addition, the coexistence of both hypermethylated and hypomethylated genes in different chronic pain conditions suggests a focused regulatory mechanism rather than a uniform change in methylation levels. While DNA methylation reprogramming does not always correspond with gene expression changes, the methylation patterns of pain-related genes can significantly influence the pain phenotype. Abbreviation: ACC, anterior cingulate cortex; M1, primary motor cortex; PFC, prefrontal cortex; S1, primary somatosensory cortex.

In patients with chronic musculoskeletal pain [24,120] and fibromyalgia [23,104], differentially methylated regions associated with pain were enriched across neurological pathways, such as GABA receptor signalling and MAPK signalling pathways [24,120]. Both pathways play critical roles in the development and maintenance of central sensitization [136]. Disruptions in GABAergic inhibition [137] and activation of MAPK pathways [65] both contribute to neuronal hyperexcitability and persistent pain. Consistently, in neuropathic pain models, increased DNA methylation following nerve injury may impair the descending inhibitory nociceptive modulation system by downregulating α_5_-GABA(A) receptor, which diminishes the inhibitory action of GABAergic neurons [138], thereby contributing to central sensitization and enhanced chronic pain states (Figure 2).

We previously reported that patients with chronic fatigue syndrome and comorbid fibromyalgia exhibited lower DNA methylation levels in the BDNF gene compared to healthy individuals, correlating with increased serum BDNF levels and hyperalgesia [20]. Similarly, high levels of biopsychosocial complexity were also linked to lower DNA methylation levels of BDNF, leading to gene upregulation in patients with chronic musculoskeletal pain [21]. Preclinical studies have shown that TET-dependent DNA demethylation promotes 5-hmC enrichment at the BDNF promoter, inducing BDNF overexpression and neuronal hyperexcitability after SNL, while inhibition of TET expression reversed these effects and alleviated nociceptive hypersensitivity [97].

In any chronic disease, identifying perpetuating factors is crucial for identifying therapeutic targets to develop preventive strategies. BDNF, a key regulator of neuroplasticity, has been proposed to play a pro-nociceptive role in mediating central sensitization [139]. Human studies consistently report higher cerebrospinal fluid [140], plasma [141,142,143], and serum [144,145,146,147] levels of BDNF in patients with chronic pain compared to healthy individuals, with these levels positively correlating with more severe pain symptoms. DNA hypomethylation tends to enhance excitatory synaptic transmission and cortical excitability by upregulating BDNF expression, contributing to central sensitization. This suggests that persistent painful stimuli at an early stage may trigger DNA methylation reprogramming, thereby promoting maladaptive transcriptional processes and central sensitization which facilitate the transition from acute to chronic pain. Notably, if this indirect sensitization occurs, DNA methylation manipulation might also reverse pro-nociceptive epigenetic marks, thereby reducing pain.

## 7. DNA Methylation Patterns in Chronic Pain: Epigenetic Interventions in a Broader Picture

The exploration of DNA methylation’s role in chronic pain is shedding light on the mechanisms of pain progression and unveiling new therapeutic possibilities, especially given the generally poor efficacy of traditional analgesics such as opioids and NSAIDs. Many genes with differential DNA methylation have been identified, playing crucial roles in nociceptive pathways with either pro- or anti-nociceptive effects. These genes may play upstream roles in the hierarchy of epigenetic events involved in chronic pain, supporting a causal role. However, the specific genes or gene networks that could be targets to reverse the epigenetic landscape associated with chronic pain remain underexplored. DNA methylation modifications within gene networks can influence the activity of associated cellular pathways, contributing to the development of chronic pain. This complex landscape of changes poses challenges for establishing causality and designing interventions to reverse chronic pain. Although rather non-specific in nature, a potential strategy involves targeting entire pathways through therapeutic combinations that focus on “hub” proteins, such as DNMTs and TET enzymes, rather than individual candidate genes.

There is limited preclinical evidence to suggest that interventions targeting these enzymes might be effective for treating chronic pain in animal models (Table 2). For instance, the nucleoside analogues 5-azacytidine and decitabine (5-aza-deoxycytidine) are established inhibitors of DNMTs, used in clinical trials to reverse epigenetic mutations in cancer and myelodysplastic syndrome patients [148,149]. Despite initial challenges with response rates and high toxicity, these compounds have shown promise in pain management contexts, such as animal models for osteoarthritis [150] and cancer-related pain [151]. Both 5-azacytidine [75,76] and decitabine [138,152] have the potential to restore the expression of hypermethylated/silenced genes and reduce pain. However, we are unaware of studies exploring the potential of these compounds in patients with chronic pain.

Additionally, RG108 effectively blocks DNMTs’ active sites. In neuropathic pain models, RG108 administration has been shown to block the increase in DNA methylation of pain-related genes by downregulating DNMT1 and DNMT3a, thereby alleviating pain symptoms [47,50,153]. Moreover, chronic systemic administration of SAM has been observed to alleviate SNI-induced nociceptive hypersensitivity, pain avoidance behaviours, and cognitive deficits. SAM also partially reverses the reduction in global DNA methylation induced by SNI [122,154]. Marketed as a dietary supplement, SAM may offer pain relief by reducing inflammation and exerting direct analgesic effects [155,156,157]. Again, studies exploring the potential of these compounds in patients with chronic pain are needed.

Epigenetic modifications of gene expression in response to environmental changes or external stimuli are dynamic and reversible [123]. This aligns with findings in other fields where reversing disease-specific DNA methylation patterns follows successful conservative treatment [158,159,160]. However, while these interventions can modify pain-associated differential DNA methylation, they generally do not restore all alterations to a pre-chronic pain state.

Although several novel DNMT inhibitors have been identified, the number of such compounds remains limited. For many of these compounds, DNA methylation inhibition has been identified as a secondary characteristic, raising concerns about their specificity [161]. For instance, while pharmacological inhibition of DNMTS offers new therapeutic avenues for cancer treatment, the lack of specificity hampers the effectiveness of these compounds. Advances in understanding the functional properties of these enzymes will pave the way for more targeted drug design. The strategic development of DNMT inhibitors could be a feasible alternative, and the creation of molecular models for screening will aid in the identification of more specific molecules. Future research is expected to deepen our knowledge of the activity and structure of DNMTs and TET enzymes, facilitating the creation of targeted inhibitors.

## 8. Conclusions and Future Direction

Biologically, an organism’s reaction to external stimuli is reflected through epigenetic modifications. These alterations can affect brain activity, leading to behavioural changes [162]. Acute pain after injury may trigger rapid and persistent DNA methylation reprogramming that evolves as pain becomes chronic. DNA methylation reprogramming occurs early during the transition from acute to chronic pain, suggesting its potential causal role. Over the past decade, the concept of neuro-epigenetics [163] has highlighted the importance of epigenetic processes in neuro-adaptive phenomena, including neuronal differentiation, synaptic plasticity, memory consolidation, and cognitive adaptability [164,165,166,167,168]. Therefore, modifications in DNA methylation may represent one mechanism in the transition of pain from an acute sensation to the pathological states of neuroinflammation, central sensitization, and, eventually, chronic pain [14].

The intricate relationship between DNA methylation and gene expression is a cornerstone in the regulation of pain. DNMTs are responsible for adding methyl groups to DNA, which can interfere with gene transcription by blocking the binding of transcription factors to gene promoters. Conversely, TET enzymes facilitate gene expression through demethylation. In addition, transcriptional regulators such as MBD proteins and MeCP2 are indispensable for modulating gene transcription. However, interpretation of DNA methylation changes warrants caution, as they do not always mirror transcriptional activity. DNA methylation can occur across various genomic regions, and not all genes are equally regulated by CpG islands [169]. Moreover, DNA methylation does not have a linear relationship with gene expression; other epigenetic mechanisms such as histone acetylation and non-coding RNAs also play significant roles. Hence, future research should integrate gene expression data for a more comprehensive analysis.

In addition, current evidence highlights that broader epigenetic interventions, beyond single gene or protein targets, are required. The strategic development of DNMT inhibitors could be a feasible alternative, and the creation of molecular models for screening will aid in the identification of more specific molecules. Further research should delve into how chronic pain is embedded in the epigenome and identify epigenetic markers for therapeutic strategies.

Finally, while associations between DNA methylation changes and pain have been established, several critical questions remain unanswered:

(i) Causal Relationship: Is there a causal relationship between DNA methylation and the occurrence of chronic pain in humans? Do DNA methylation changes result from chronic pain or precede its emergence? Are DNA methylation alterations merely stochastic footprints downstream of underlying pain neuropathology in patients with chronic pain conditions?
(ii) Temporal Dynamics: How rapidly do DNA methylation changes occur, and are they persistent during chronic pain? How does epigenetic dysregulation at the onset of acute pain progress as pain transitions from acute to chronic in humans?
(iii) Gene-Specific vs. Domain-Wide Changes: What is the extent of DNA methylation alterations in the brain after chronic pain induction, and are these changes specific to pain-related genes or broader domains and/or gene families?
(iv) Translation to Clinical Practise: How can we translate epidemiological findings to the clinic? How can findings at the population level be applied to individual patients? What is the potential impact on pain perception, and to what extent can we influence this through therapy?
(v) Therapeutic Potential: is DNA methylation reversible and targetable by existing or proposed treatments? Which of these processes should be targeted for therapeutic intervention?
